# Orchestrating inflammation: non-coding RNAs as master regulators of macrophage function in chronic obstructive pulmonary disease-an update

**DOI:** 10.3389/fimmu.2025.1679730

**Published:** 2025-10-28

**Authors:** Xinyu Yong, Xian Luo, Xiaobing Chen, Chengxiu Yu

**Affiliations:** ^1^ Department of Respiratory and Critical Care Medicine, The Affiliated Hospital of North Sichuan Medical College, Nanchong, Sichuan, China; ^2^ North Sichuan Medical College, Nanchong, Sichuan, China

**Keywords:** chronic obstructive pulmonary disease, non-coding RNAs, macrophages, inflammation, biomarkers

## Abstract

Chronic obstructive pulmonary disease (COPD) represents a major global health burden, characterized by dysregulated macrophage function and persistent inflammation. Non-coding RNAs (ncRNAs), including microRNAs (miRNAs), long non-coding RNAs (lncRNAs), and circular RNAs, have emerged as critical orchestrators of macrophage polarization and inflammatory responses in COPD pathogenesis. This comprehensive review synthesizes current evidence demonstrating how ncRNA-macrophage regulatory axes drive disease progression. Pro-inflammatory miRNAs promote pathological M1 polarization through NF-κB and STAT3 pathways, while protective miRNAs facilitate inflammation resolution. LncRNAs exhibit sophisticated regulatory mechanisms through transcriptional scaffolding and competitive endogenous RNA networks. Clinical studies have successfully translated these mechanistic insights, establishing diagnostic biomarkers and therapeutic targets in human COPD patients. Despite significant progress, challenges remain including methodological heterogeneity, limited understanding of integrated regulatory networks, and clinical translation barriers. Future directions emphasize precision medicine approaches through ncRNA-based diagnostics and combination therapeutics. The evidence strongly supports the therapeutic potential of targeting ncRNA-macrophage regulatory axes, offering transformative opportunities for personalized COPD management and improved patient outcomes.

## 1 Introduction

Chronic obstructive pulmonary disease (COPD) represents one of the major global health burden diseases, with statistics indicating that more than 174 million people worldwide were affected and represents the fourth leading cause of death in the United States (1). Despite significant advances in understanding COPD pathogenesis, the disease remains characterized by persistent inflammation and progressive airflow limitation. This results in substantial morbidity and mortality (2). The complex pathophysiology of COPD involves multiple cellular and molecular mechanisms, including abnormal inflammatory responses, impaired tissue repair, and dysregulated immune cell function (1, 2). Recent insights have moved beyond the traditional understanding of COPD as simply accelerated lung function decline. Current research now recognizes distinct disease trajectories and early pathobiological changes that precede clinically apparent disease (3). Understanding these complex pathophysiological mechanisms is crucial for developing targeted therapeutic interventions.

Central to these pathophysiological processes, macrophages play a pivotal role in COPD pathogenesis, serving as key orchestrators of both innate and adaptive immune responses in the lung (4). Importantly, the pulmonary macrophage compartment comprises functionally and anatomically distinct populations: alveolar macrophages (AMs) residing in airspaces serve as sentinel cells for pathogen recognition and particle clearance, while interstitial macrophages (IMs) within the lung parenchyma primarily mediate tissue remodeling and stromal interactions (4, 5). Furthermore, these populations differ in developmental origin, with tissue-resident macrophages (embryonic-derived, self-renewing) displaying distinct transcriptional programs compared to recruited monocyte-derived macrophages that infiltrate during inflammation (5). In healthy individuals, these macrophage subsets maintain pulmonary homeostasis through coordinated phagocytosis, pathogen clearance, and tissue repair functions (5). However, in COPD pathogenesis, macrophage function becomes severely dysregulated across these distinct populations. This dysregulation is characterized by altered activation states, enhanced pro-inflammatory cytokine production, impaired phagocytic capacity, and defective efferocytosis (6). Critically, COPD significantly alters the balance between tissue-resident and recruited macrophages, with increased monocyte infiltration potentially creating distinct molecular signatures and functional properties (5, 6). The polarization of macrophages toward pro-inflammatory M1 phenotypes, coupled with impaired M2-mediated tissue repair mechanisms, contributes to persistent inflammation and progressive tissue destruction observed in COPD (7, 8).

The regulation of these complex macrophage functions involves multiple molecular mechanisms, among which non-coding RNAs (ncRNAs), including microRNAs (miRNAs), long non-coding RNAs (lncRNAs), and circular RNAs (circRNAs), have emerged as critical regulators of gene expression and cellular function across various biological processes (9). These molecular regulators exert their effects through diverse mechanisms, including post-transcriptional gene silencing, chromatin modification, and competitive endogenous RNA (ceRNA) networks (9, 10). In the context of immune regulation, ncRNAs have been shown to control macrophage activation, polarization, and functional responses, thereby influencing inflammatory processes and disease outcomes (11, 12). Dysregulation of ncRNA expression patterns has been implicated in various respiratory diseases, suggesting their potential as both biomarkers and therapeutic targets (13).

The intersection of ncRNA biology and macrophage function has revealed complex regulatory networks that govern immune responses in health and disease (11, 12). Emerging evidence demonstrates that specific ncRNA-macrophage regulatory axes play crucial roles in COPD pathogenesis, influencing inflammation resolution, tissue remodeling, and disease progression (14, 15). Studies in asthma and other inflammatory conditions have shown that ncRNAs can either promote or suppress macrophage-mediated inflammation, depending on the specific molecular context and disease stage (16, 17). Despite these advances, the precise mechanisms by which ncRNAs regulate macrophage function in COPD, and the therapeutic potential of targeting these pathways, remain incompletely characterized.

This comprehensive review aims to synthesize current knowledge on ncRNA-macrophage regulatory axes in COPD, providing a systematic analysis of molecular mechanisms, clinical implications, and therapeutic prospects. We will examine the latest advances in understanding how different classes of ncRNAs modulate macrophage behavior in COPD pathogenesis, evaluate the translational potential of these findings, and identify key research gaps that warrant further investigation. By elucidating these complex regulatory networks, this review seeks to inform the development of novel precision medicine approaches for COPD management and highlight promising avenues for future therapeutic intervention.

## 2 Molecular mechanisms of ncRNA regulation of macrophage function

### 2.1 MiRNA-mediated macrophage regulation

MiRNAs represent the most extensively characterized class of ncRNAs in macrophage biology, functioning primarily through post-transcriptional gene silencing mechanisms (18). The canonical miRNA pathway involves binding to complementary sequences in the 3’ untranslated regions of target messenger RNAs (mRNAs), leading to mRNA degradation or translational repression (19). In macrophages, specific miRNAs have been identified as critical regulators of activation and polarization states, with distinct expression patterns associated with M1 and M2 phenotypes (20).

Several miRNAs have emerged as key modulators of macrophage polarization through targeting of transcription factors and signaling molecules. For instance, exosomes derived from adipose-derived stem cells can modulate M1/M2 macrophage phenotypic polarization through regulation of the *miR-451a*/macrophage migration inhibitory factor axis, thereby promoting bone healing (20).

Similarly, in hepatic fibrosis, various miRNAs (*miR-206, miR-26a, miR-155*, and *miR-148a*) exert anti-fibrotic effects by regulating oxidative stress, modulating cytokine secretion, and promoting CD8+ T cell recruitment (21). These regulatory networks exemplify the fine-tuned control that miRNAs exert over macrophage functional states.

The therapeutic targeting of miRNAs in macrophages has shown promise in preclinical studies. MiRNA mimics and antagomirs have been successfully employed to modulate macrophage polarization in various disease models (22, 23). However, challenges remain in achieving cell-type-specific delivery and avoiding off-target effects, particularly given the widespread expression of many miRNAs across different cell types (24).

### 2.2 LncRNA regulatory networks

While miRNAs primarily regulate gene expression at the post-transcriptional level, lncRNAs exhibit more diverse regulatory mechanisms in macrophage regulation, including transcriptional modulation through DNA interaction, ceRNA/miRNA sponge activity, protein binding, and micropeptide encoding (25). Unlike miRNAs, lncRNAs can interact directly with chromatin-modifying complexes, transcription factors, and other regulatory proteins to influence gene expression at multiple levels (25, 26). This versatility allows lncRNAs to orchestrate complex gene expression programs associated with macrophage differentiation and activation.

Several lncRNAs have been identified as important regulators of macrophage polarization. For instance, lncRNA *MIAT* downregulates interleukin-1β (IL-1β) and tumor necrosis factor alpha (TNF-α) to suppress macrophage inflammation (27). However, this anti-inflammatory effect is inhibited by ATP-induced NLRP3 inflammasome activation, highlighting the context-dependent nature of lncRNA *MIAT* function (27). Similarly, research has shown that human lncRNA *SUGCT-AS1* can directly bind to heterogeneous nuclear ribonucleoprotein U (hnRNPU) and regulate its nuclear-cytoplasmic translocation. This translocation of hnRNPU modulates macrophage inflammation by altering the ratio of mucosa-associated lymphoid tissue lymphoma translocation protein 1 (MALT1) isoforms through regulation of MALT1 alternative splicing (28). Interestingly, macrophages themselves can secrete corresponding lncRNAs. Studies have revealed that M2 macrophage exosome-derived lncRNA *AK083884* protects mice against coxsackievirus B3-induced viral myocarditis through regulating pyruvate kinase M2/hypoxia-inducible factor-1alpha axis-mediated macrophage metabolic reprogramming (29). These findings unveil the intricate interplay between lncRNAs and macrophages.

### 2.3 CircRNA-mediated regulation

Building upon the diverse regulatory mechanisms of lncRNAs, circRNAs have emerged as important regulators of macrophage function, primarily through their role as miRNA sponges in ceRNA networks (30). Recent studies have identified several circRNAs that modulate macrophage polarization. For example, studies have demonstrated that exosome-based mitochondrial delivery of circRNA *mSCAR* can promote macrophage polarization toward the M2 subtype, attenuate systemic inflammation, and reduce mortality, thereby alleviating sepsis (31). Interestingly, additional research has shown that macrophage uptake of circRNAs is rapid, energy-dependent, and saturable. CircRNA uptake can lead to translation of coding sequences and antigen presentation, and the internalization pathway can influence immune activation following circRNA uptake. Further investigation revealed that macrophage scavenger receptor 1, toll-like receptors (TLRs), and mammalian target of rapamycin signaling are key regulators of receptor-mediated circRNA phagocytosis (32). These findings demonstrate the emerging importance of circRNAs in macrophage biology and their potential as therapeutic targets.

Moreover, the regulatory networks involving circRNAs are often complex, with individual circRNAs capable of binding multiple miRNAs and influencing numerous downstream targets (30, 33). This network complexity provides both opportunities and challenges for therapeutic intervention, as modulation of a single circRNA may have widespread effects on cellular function.

### 2.4 Integrated regulatory networks and cross-talk

The regulation of macrophage function involves complex interactions between different classes of ncRNAs, creating multilayered regulatory networks (17, 21). These networks exhibit significant cross-talk, with miRNAs, lncRNAs, and circRNAs often competing for binding sites and regulatory proteins (30, 33). Understanding these integrated networks is crucial for developing effective therapeutic strategies targeting ncRNA-macrophage axes.

Computational approaches have revealed extensive ceRNA networks in macrophages, with hundreds of lncRNAs and circRNAs predicted to interact with key regulatory miRNAs (34). Experimental validation of these networks has confirmed the functional importance of many predicted interactions, highlighting the coordinated nature of ncRNA-mediated regulation (35). The dynamic nature of these networks allows for context-dependent regulation, with different stimuli activating distinct regulatory modules.

The molecular mechanisms underlying ncRNA-mediated macrophage regulation demonstrate remarkable complexity and sophistication. MiRNAs primarily function through post-transcriptional gene silencing, targeting key transcription factors and signaling molecules that determine macrophage activation states. LncRNAs exhibit more diverse regulatory mechanisms, including chromatin modification, transcriptional scaffolding, and ceRNA network formation, providing multilayered control over macrophage phenotypes. CircRNAs, with their stable covalent structure, function predominantly as miRNA sponges, adding another dimension to regulatory networks. The extensive cross-talk between these ncRNA classes creates integrated regulatory circuits that enable context-dependent, fine-tuned control of macrophage responses. Understanding these interconnected mechanisms is essential for developing targeted therapeutic strategies that can modulate specific pathogenic pathways while preserving beneficial macrophage functions in respiratory diseases.

## 3 COPD pathogenesis and the role of macrophages

### 3.1 Overview of COPD pathogenesis

COPD pathogenesis involves complex interplay among genetic susceptibility, environmental exposures, and abnormal inflammatory responses. These factors lead to progressive airway and parenchymal destruction (1, 2). The disease is characterized by two major pathological features: emphysema, involving destruction of alveolar walls and loss of elastic recoil, and chronic bronchitis, characterized by airway inflammation, mucus hypersecretion, and airway wall thickening (2, 3). These pathological changes result from an imbalance between tissue destruction and repair processes, with inflammatory cells playing central roles in disease initiation and progression (4).

Cigarette smoke exposure represents the primary risk factor for COPD development, triggering a cascade of cellular and molecular events that ultimately lead to tissue damage (1, 2). The initial response to smoke exposure involves activation of epithelial cells and resident macrophages, leading to recruitment of inflammatory cells and production of pro-inflammatory mediators. Over time, this acute inflammatory response becomes dysregulated, resulting in chronic inflammation that persists even after smoking cessation (4–6).

Recent advances in understanding COPD pathogenesis have revealed the importance of early disease processes, including epigenetic modifications, altered lung development, and dysregulated immune responses (1–3). These insights have shifted the focus toward identifying early biomarkers and therapeutic targets that could prevent or slow disease progression before significant structural damage occurs (36).

### 3.2 Macrophage dysfunction in COPD

The pulmonary macrophage compartment encompasses distinct subsets with specialized functions that become differentially dysregulated in COPD (5). AMs, which reside in the airway lumen and alveolar spaces, constitute the predominant lung macrophage population and serve as the first line of defense against inhaled pathogens and particles. In contrast, IMs populate the lung parenchyma and primarily regulate tissue homeostasis and remodeling. These populations also differ by developmental origin: tissue-resident macrophages are embryonically derived and self-renewing, whereas monocyte-derived macrophages are recruited from circulating blood during inflammation. In healthy lungs, this heterogeneous macrophage network maintains homeostasis through coordinated clearance of inhaled particles, pathogens, and apoptotic cells (4–6). A key feature of macrophage dysfunction in COPD is impaired phagocytosis, particularly affecting clearance of bacteria and apoptotic cells (6). This defective function contributes to increased susceptibility to respiratory infections and persistent inflammation (37). Studies have demonstrated that alveolar macrophages from COPD patients exhibit reduced phagocytic capacity compared to healthy controls, with this impairment correlating with disease severity (38).

**Figure 1:**
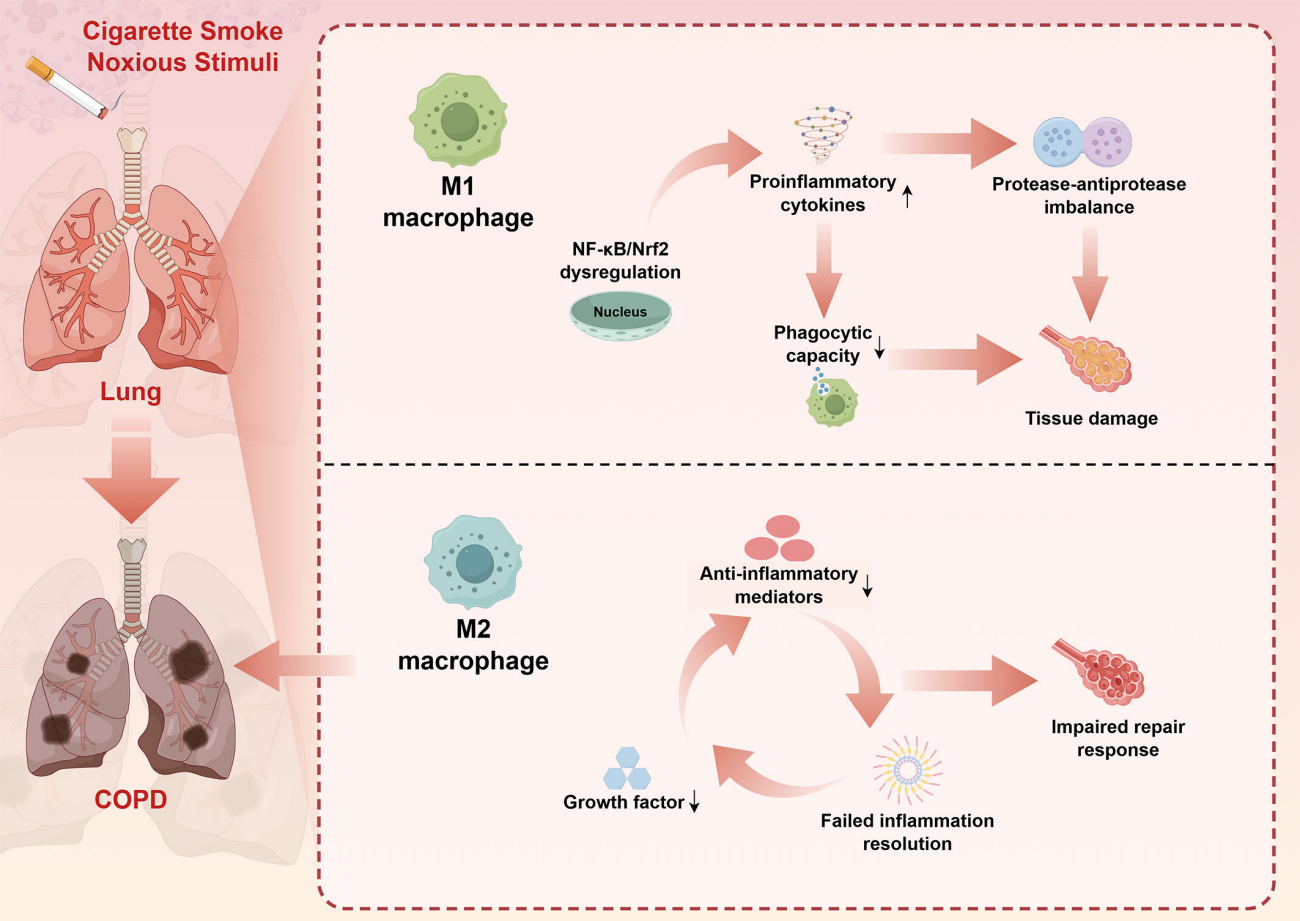
Macrophage dysfunction in COPD pathogenesis: A simplified mechanistic overview. This schematic diagram synthesizes key pathophysiological mechanisms derived from comprehensive literature review (Sections 3.1-3.5). Cigarette smoke and other noxious stimuli trigger dysregulated macrophage responses in the lung, leading to COPD development. The upper pathway illustrates M1 macrophage activation characterized by NF-κB/Nrf2 dysregulation, resulting in enhanced proinflammatory cytokine production, protease-antiprotease imbalance, impaired phagocytic capacity, and subsequent tissue damage. The lower pathway demonstrates M2 macrophage dysfunction, featuring reduced anti-inflammatory mediator production, decreased growth factor secretion, and failed inflammation resolution, ultimately contributing to impaired repair responses. Bidirectional arrows indicate the cyclical nature of inflammatory processes. This imbalanced M1/M2 polarization perpetuates chronic inflammation and progressive tissue destruction characteristic of COPD pathogenesis. The figure was created using Figdraw (www.figdraw.com). COPD, chronic obstructive pulmonary disease; NF-κB, nuclear factor-kappa B; Nrf2, NF-E2-related factor 2.

The polarization state of macrophages is also significantly altered in COPD, with evidence suggesting a predominance of M1-like inflammatory phenotypes and impaired M2-mediated repair responses (6). This imbalanced polarization contributes to sustained inflammation and defective tissue repair, perpetuating the cycle of tissue destruction characteristic of COPD (3). The molecular mechanisms underlying this polarization imbalance involve dysregulation of key transcription factors, including nuclear factor-kappaB (NF-κB), phosphatidylinositol-3-kinase (PI3K)-protein kinase B (AKT), and NF-E2-related factor 2 (4, 14, 39). A brief summary of the influence of macrophages on the disease progression of COPD is presented in **Figure 1**.

### 3.3 Protease-antiprotease imbalance

Macrophages contribute significantly to the protease-antiprotease imbalance that is central to emphysema development (40). Activated macrophages release various proteases, including matrix metalloproteinases (MMPs), neutrophil elastase, and cathepsins, which degrade extracellular matrix components (41). In COPD, this proteolytic activity is enhanced while antiprotease defenses are overwhelmed, leading to progressive tissue destruction (42).

Multiple MMPs have been demonstrated to participate in regulating COPD development and progression, and may serve as potential diagnostic markers for COPD patients. For instance, studies have found elevated serum MMP-9 and tissue inhibitor of metalloproteinases-1 (TIMP-1) levels, as well as increased MMP-9/TIMP-1 ratios in COPD patients. The imbalance between MMP-9 and TIMP-1 in COPD patients favors a pro-proteolytic environment, collectively highlighting the importance of the MMP-9/TIMP-1 ratio as a potential biomarker for COPD diagnosis and severity assessment (43).

Additionally, other research has demonstrated that deflamin, a protein component extracted from lupin, can attenuate lung tissue damage in ozone-induced COPD mouse models by regulating MMP-9 catalytic activity (44). Notably, ncRNAs have also been proven to participate in regulating MMP expression. For example, the lncRNA *TP73-AS1*/*miR-539*/MMP-8 axis regulates M2 macrophage polarization in hepatocellular carcinoma through transforming growth factor-beta1 signaling (45). These findings provide further evidence for the interplay between ncRNAs and macrophages in COPD pathogenesis, while also highlighting the crucial role of protease dysregulation, represented by MMPs, in COPD pathogenesis.

### 3.4 Oxidative stress and macrophage function

Chronic exposure to cigarette smoke leads to sustained oxidative stress in the lung, significantly affecting macrophage function (6). Reactive oxygen species generated by activated macrophages contribute to tissue damage while also serving as signaling molecules that modulate inflammatory responses. The antioxidant capacity of macrophages becomes overwhelmed in COPD, leading to a pro-oxidant environment that perpetuates inflammation and tissue damage (46).

Oxidative stress affects multiple aspects of macrophage function, including phagocytosis, cytokine production, and cell survival. The dysregulation of antioxidant enzymes and reduced glutathione levels in COPD macrophages contribute to this oxidative burden (5, 6). Understanding the molecular mechanisms linking oxidative stress to macrophage dysfunction provides insights into potential therapeutic approaches targeting this pathway (1).

### 3.5 Macrophage-mediated tissue remodeling

Beyond their role in inflammation, macrophages are crucial mediators of tissue remodeling processes in COPD. The balance between tissue destruction and repair is significantly disrupted in COPD, with macrophages contributing to both processes (2, 3). M2 macrophages typically promote tissue repair through production of growth factors and anti-inflammatory mediators, but this repair response is often inadequate or misdirected in COPD (47).

The interaction between macrophages and other lung cells, including fibroblasts, epithelial cells, and smooth muscle cells, is critical for proper tissue remodeling (48). Dysregulated communication between these cell types contributes to airway wall thickening, mucus gland hyperplasia, and abnormal repair responses observed in COPD (4–6). Understanding these cellular interactions provides insights into the complex pathophysiology of COPD and potential targets for therapeutic intervention.

In summary, COPD pathogenesis fundamentally involves dysregulated macrophage function at multiple levels. Chronic exposure to cigarette smoke and other noxious stimuli transforms protective alveolar macrophages into drivers of disease progression through impaired phagocytosis, aberrant M1/M2 polarization, excessive protease release, and defective tissue repair responses. The resulting protease-antiprotease imbalance, coupled with oxidative stress and abnormal tissue remodeling, creates a self-perpetuating cycle of inflammation and tissue destruction. The complex interactions between macrophages and other lung cells, including epithelial cells, fibroblasts, and immune cells, further amplify pathological processes. These multifaceted macrophage dysfunctions provide numerous potential intervention points for therapeutic targeting, emphasizing the central role of macrophage biology in COPD pathogenesis and the critical need to understand how ncRNAs regulate these processes. In the following sections, we examine how ncRNAs specifically modulate macrophage function in COPD, progressing from preclinical mechanistic studies to clinical translation.

## 4 NcRNA-macrophage regulatory axis in COPD: research progress

### 4.1 Preclinical studies

Preclinical investigations have identified distinct functional categories of ncRNAs that regulate macrophage polarization and inflammatory responses in COPD models. These studies reveal three major regulatory patterns: (1) pro-inflammatory ncRNAs that activate NF-κB and STAT3 pathways to drive M1 polarization; (2) protective ncRNAs that inhibit inflammation and prevent emphysema formation; and (3) metabolic ncRNAs that modulate lipid metabolism and cellular stress responses. The following sections synthesize key mechanistic insights, with comprehensive details provided in **Tables 1**, **2**.

**Table 1:** Preclinical studies of miRNAs in macrophage regulation in COPD.

miRNA	Expression pattern	Target/mechanism	Macrophage effect	Functional outcome	Reference
Pro-inflammatory miRNAs					
*miR-21*	↑ in CS-exposed lungs and macrophages	SATB1/S100A9/NF-κB axis	Enhanced inflammatory activation	Promotes lung inflammation and emphysema; correlates with reduced lung function	(14)
*miR-125a-5p*	↑ in CSE-treated epithelial cell exosomes	IL1RN via MyD88/NF-κB pathway	Promotes M1 polarization	Facilitates epithelium-macrophage inflammatory crosstalk	(15)
*miR-221-3p*	↑ in CS-induced epithelial exosomes	SOCS3 via STAT3 pathway	Facilitates M1 polarization	Promotes cigarette smoke-induced inflammation	(49)
*miR-27-3p*	↑ in CS/LPS-exposed alveolar macrophages	PPARγ/TLR2/4 pathway	Promotes macrophage activation and M1 polarization	Regulates TLR-dependent inflammatory responses	(50)
*miR-155*	↑ in CS-exposed lungs and alveolar macrophages	Rictor/mTORC2/RhoA pathway	Enhanced phagocytosis but promotes inflammation	Paradoxical: improves phagocytosis while promoting emphysema	(51, 52)
Protective miRNAs					
*miR-195-5p*	↓ in COPD	Siglec1/NF-κB pathway	Inhibits pro-inflammatory cytokine production	Protective against COPD development	(53)
*miR-let-7c*	↓ in COPD patients and CS-exposed mice	IL-6/STAT3 pathway	Inhibits M2 polarization (paradoxical COPD protection)	Challenges conventional M2 protective role	(54)
*miR-146a*	↑ in response to elastase/CS (protective response)	Inflammatory response regulation	Protects against abnormal inflammatory responses	Prevents emphysema formation and inflammation	(55)
*miR-513a-5p*	↓ in COPD	TFR1-dependent signaling	Regulates airway mucous cell hyperplasia and macrophage activation	Controls MUC5AC hypersecretion and inflammation	(56)
*miR-344b-1-3p*	↑ in COPD alveolar macrophages	TLR2 pathway	Negatively regulates TLR2 signaling and inflammation	Modulates inflammatory responses to prevent IPA	(57)
Metabolic and cellular stress response miRNAs					
*miR-103a*	↓ in smokers and COPD patients	Low-density lipoprotein receptors	Impaired lipid metabolism leading to lipid-laden macrophages	Contributes to altered macrophage phenotype	(58)
*miR-451b*	↓ (regulated by p300)	RhoA/ROCK2 pathway	Reduces inflammatory cytokine release and cell migration	Protects against CSE-induced cellular stress	(59)
*miR-486-5p*	↑ in COPD patients and smokers	HAT1/TLR4 pathway	Enhanced inflammatory response	Promotes TLR4-triggered inflammation	(60)
*miR-150*	↓ in CS-exposed tissues	p53/NF-κB pathway	Protects against CS-induced inflammation and apoptosis	Prevents lung inflammation and epithelial cell death	(61)
*miR-1260*	↑ via Siglec1 pathway	IκBα degradation	Enhanced inflammatory cytokine production	Amplifies NF-κB-mediated inflammation	(62)
Multi-component regulatory systems					
*miR-301a-5p*	↓ in acute COPD exacerbation	CXCL12/CXCR4 via MBD2 regulation	Affects macrophage migration through MEK/AKT signaling	Regulates acute exacerbations	(63)
*miR-380*	↑ in COPD macrophage-derived exosomes	CFTR pathway	Promotes epithelial cell proliferation and inflammatory responses	Mediates macrophage-epithelial communication	(64)
*miR-124-3p*	↑ with exercise training	ERN1 pathway	Inhibits M1 macrophage activation	Exercise-mediated anti-inflammatory effects	(65)
Omics and systems biology					
*miR-29a-3p, miR-1307-5p, miR-21-3p, miR-27b-3p*	↑ in CSE-treated epithelial exosomes	PI3K-Akt and MAPK pathways	Promotes both M1 and M2 macrophage polarization	Comprehensive macrophage reprogramming	(66)
Multiple miRNAs	Global ↓ due to DICER dysfunction	DICER SUMOylation	Impaired miRNA processing affects entire regulatory network	Broad dysregulation of macrophage responses	(67)
*miR6511a-5p*	Involved in miRNA-mRNA interactions	NT5E	Affects cell-cell contact and immune cell activation in alveolar macrophages	Influences cellular homeostasis and T lymphocyte activation	(68)

AKT, protein kinase B; CFTR, cystic fibrosis transmembrane conductance regulator; CS, cigarette smoke; CSE, cigarette smoke extract; CXCL12, C-X-C motif chemokine ligand 12; CXCR4, C-X-C motif chemokine receptor 4; DICER, double-stranded RNA-specific endoribonuclease; ERN1, endoplasmic reticulum to nucleus signaling 1; HAT1, histone acetyltransferase 1; IκBα, nuclear factor of kappa light polypeptide gene enhancer in B-cells inhibitor alpha; IL-6, interleukin-6; IL1RN, interleukin-1 receptor antagonist; IPA, invasive pulmonary aspergillosis; LPS, lipopolysaccharide; MAPK, mitogen-activated protein kinase; MBD2, methyl-CpG-binding domain protein 2; MEK, mitogen-activated protein kinase kinase; miR/miRNA, microRNA; MUC5AC, mucin 5AC; mTORC2, mechanistic target of rapamycin complex 2; MyD88, myeloid differentiation primary response 88; NF-κB, nuclear factor kappa B; NT5E, 5’-nucleotidase ecto; p53, tumor protein 53; PI3K, phosphoinositide 3-kinase; PPARγ, peroxisome proliferator-activated receptor gamma; RhoA, ras homolog family member A; Rictor, rapamycin-insensitive companion of mechanistic target of rapamycin; ROCK2, rho associated coiled-coil containing protein kinase 2; S100A9, S100 calcium binding protein A9; SATB1, special AT-rich sequence-binding protein 1; Siglec1, sialic acid-binding immunoglobulin-like lectin 1; SOCS3, suppressor of cytokine signaling 3; STAT3, signal transducer and activator of transcription 3; TFR1, transferrin receptor 1; TLR, toll-like receptor; ↑, upregulated; ↓, downregulated.

**Table 2:** Preclinical studies of lncRNAs in macrophage regulation in COPD.

lncRNA	Expression change	Target/mechanism	Macrophage effects	Functional outcomes	Reference
Pro-inflammatory lncRNAs					
LncRNA *MEG3*	↑ in CSE-treated epithelial cell exosomes	TREM-1 upregulation via m6A methylation (SPI1/METTL3 pathway)	Promotes M1 polarization and pyroptosis	Enhances inflammatory responses and cell death programs	(69)
LncRNA *MIR155HG*	↑ in GM-CSF-induced macrophages from COPD patients	Direct regulation of M1/M2 balance	Promotes M1 polarization and pro-inflammatory cytokine release; inhibits M2 development	Contributes to smoke-related COPD through *miR-128-5p*/BRD4 axis	(70, 71)
LncRNA *TBX2-AS1*	↑ in serum-derived exosomes from COPD patients	*miR-423-5p*/*miR-23b-3p* axis (targets CELSR2 and NEK6)	Alters M1/M2 macrophage ratios: promotes M1, inhibits M2	Exacerbates COPD progression through systemic exosomal delivery	(72)
Regulatory and protective lncRNAs					
LncRNA *Cox2*	↑ in response to CS (protective response)	Negative regulation of inflammatory gene expression	Downregulates inflammatory responses in smoke-exposed macrophages	Serves as endogenous protective mechanism	(73)
LncRNA *IL7R*	↓ in COPD patients (correlates with exacerbator phenotype)	TLR2/4-mediated chromatin remodeling	Mediates repressive chromatin states at pro-inflammatory gene promoters	Associated with acute exacerbation risk; protective against TLR-mediated inflammation	(74)
LncRNA *PPP2R5C*	↑ in pulmonary macrophages from COPD mice	IL-1β ubiquitination regulation via PP2A activity	Controls IL-1β protein levels through post-translational modification	Deficiency ameliorates emphysema and pulmonary inflammation	(75)
LncRNA *Clic5*	↓ in PM2.5-exposed alveolar macrophages	*miR-212-5p* sponging to regulate RASSF1	Functions as ceRNA to inhibit particulate matter-induced apoptosis	Protects against environmental stress-induced cell death	(76)
LncRNA 656	↑ in CSE-treated epithelial cell	Small airway epithelium analysis	TNMD correlates with activated CD4 memory T cells, M1 macrophages, and activated NK cells	Anoikis resistance contributes to COPD progression through immune microenvironment modulation	(77)

BRD4, bromodomain-containing protein 4; CELSR2, cadherin EGF LAG seven-pass G-type receptor 2; ceRNA, competitive endogenous RNA; CS, cigarette smoke; CSE, cigarette smoke extract; GM-CSF, granulocyte-macrophage colony-stimulating factor; IL-1β, interleukin-1 beta; lncRNA, long non-coding RNA; PPP2R5C, protein phosphatase 2 regulatory subunit B gamma; Clic5, chloride intracellular channel 5; IL7R, interleukin-7 receptor; m6A, N6-methyladenosine; MEG3, maternally expressed gene 3; METTL3, methyltransferase-like 3; miR, microRNA; NEK6, NIMA related kinase 6; NK, natural killer; PM2.5, particulate matter with diameter ≤2.5 micrometers; PP2A, protein phosphatase 2A; RASSF1, ras association domain family member 1; SPI1, spi-1 proto-oncogene; TLR, toll-like receptor; TNMD, tenomodulin; TREM-1, triggering receptor expressed on myeloid cells 1; ↑, upregulated; ↓, downregulated.

#### 4.1.1 MiRNA-mediated macrophage regulation in COPD

MiRNAs have emerged as pivotal regulators of macrophage function in COPD pathogenesis, with distinct miRNAs exhibiting either pro-inflammatory or protective effects. These regulatory molecules orchestrate complex signaling networks that ultimately determine macrophage polarization states and functional outcomes in the diseased lung. The preclinical research results related to miRNAs involved in macrophage regulation in COPD are summarized in **Table 1**.

Pro-inflammatory miRNAs predominantly activate NF-κB and signal transducer and activator of transcription 3 (STAT3) pathways to drive M1 polarization. *miR-21* exemplifies this pattern, promoting COPD pathogenesis through a SATB1/S100A9/NF-κB signaling axis, with expression levels inversely correlating with lung function (14). Importantly, therapeutic *miR-21* inhibition effectively suppresses inflammatory cell infiltration and improves lung function in experimental models (14). Intercellular communication amplifies these effects: epithelial cell-derived exosomal miRNAs (*miR-125a-5p, miR-221-3p*) promote M1 polarization in recipient macrophages via IL1RN/MyD88/NF-κB and SOCS3/STAT3 pathways, respectively (15, 49). Additional pro-inflammatory miRNAs including *miR-27-3p, miR-155, miR-486-5p*, and *miR-1260* similarly activate macrophages through TLR signaling, mTORC2/RhoA, and NF-κB pathways (50–52, 60, 62).

In contrast, protective miRNAs inhibit inflammation and prevent emphysema formation. *miR-195-5p* suppresses COPD development by targeting siglec1 and inactivating NF-κB signaling (53), while *miR-146a* deficiency results in increased emphysema severity and enhanced pro-inflammatory mediator production in murine models (55). Paradoxically, *miR-let-7c* inhibits M2 macrophage polarization through the IL-6/STAT3 pathway, suggesting that excessive M2 polarization may contribute to emphysema rather than repair in COPD (54). Additional protective miRNAs (*miR-513a-5p, miR-344b-1-3p, miR-150*) negatively regulate TLR signaling and inflammatory responses (56, 57, 61).

Metabolic dysregulation represents a third regulatory layer. *miR-103a* downregulation in smokers and COPD patients impairs lipid metabolism through targeting low-density lipoprotein receptors, leading to lipid-laden macrophage formation (58). *miR-451b* protects against cigarette smoke-induced cellular stress by targeting the RhoA/ROCK2 pathway, decreasing inflammatory cytokine release and suppressing macrophage migration (59). These metabolic miRNAs collectively modulate lipid metabolism, cellular stress responses, and TLR signaling pathways.

Notably, fundamental alterations in miRNA processing machinery contribute to COPD pathogenesis. Cigarette smoke exposure causes global miRNA downregulation in alveolar macrophages through DICER SUMOylation, impairing the entire miRNA regulatory network (67). This processing defect, combined with specific miRNA-mRNA interaction networks identified through transcriptomic analyses (68), highlights the coordinated dysregulation of macrophage responses in COPD. Additional regulatory miRNAs and their mechanisms are detailed in **Table 1**.

#### 4.1.2 LncRNA regulatory networks

LncRNAs exhibit diverse mechanisms of action in macrophage regulation, functioning as molecular scaffolds, transcriptional regulators, and ceRNAs. These versatile molecules orchestrate complex gene expression programs that significantly influence macrophage differentiation, activation, and functional responses in COPD. The preclinical research results related to lncRNAs involved in macrophage regulation in COPD are summarized in **Table 2**.

Pro-inflammatory lncRNAs drive M1 polarization through sophisticated molecular mechanisms. Cigarette smoke-exposed epithelial cell-derived exosomal lncRNA *MEG3* promotes M1 macrophage polarization and pyroptosis by recruiting SPI1 to activate METTL3, which increases N6-methyladenosine methylation of TREM-1 mRNA (69). LncRNA *MIR155HG* demonstrates bidirectional regulatory capacity: overexpression promotes granulocyte-macrophage colony-stimulating factor-induced M1 polarization and pro-inflammatory cytokine release, while knockdown enhances M2 development (70, 71). Serum-derived exosomal lncRNA *TBX2-AS1* further exacerbates COPD by altering M1/M2 ratios through the *miR-423-5p*/*miR-23b-3p* axis (72), illustrating systemic lncRNA-mediated regulation.

Regulatory lncRNAs provide protective or modulatory functions through distinct mechanisms. LncRNA *Cox2* serves as a negative regulator of smoke-induced inflammation, downregulating inflammatory gene expression in cigarette smoke-exposed macrophages (73). LncRNA *IL7R*, reduced in COPD patients with exacerbator phenotypes, mediates repressive chromatin states at pro-inflammatory gene promoters through H3K9ac reduction and H3K9me3/H3K27me3 increases (74). Post-translational regulation is exemplified by lncRNA *PPP2R5C*, which controls IL-1β ubiquitination through protein phosphatase 2A activity modulation (75).

The ceRNA mechanism represents a particularly important regulatory paradigm. LncRNA *Clic5* functions as a molecular sponge for *miR-212-5p*, inhibiting particulate matter-induced apoptosis in alveolar macrophages (76). This network-level regulation allows coordinated control of multiple genes involved in macrophage function, providing fine-tuned inflammatory responses. Additional regulatory lncRNAs and their mechanisms are detailed in **Table 2**.

#### 4.1.3 Multi-component regulatory systems and therapeutic implications

Systems-level analyses reveal complex ncRNA networks integrating multiple regulatory layers. Clinical correlations demonstrate that decreased *miR-301a-5p* in acute exacerbation patients regulates lung fibroblast and monocyte-derived macrophage migration through the MBD2/CXCL12/CXCR4/p-MEK/p-AKT pathway (63). The *miR-380*/CFTR axis illustrates bidirectional macrophage-epithelial communication: elevated *miR-380* in COPD macrophage-derived exosomes promotes epithelial cell proliferation, mucin expression, and inflammatory cytokine secretion (64). Non-pharmacological interventions also operate through ncRNA mechanisms, as demonstrated by exercise training-induced *miR-124-3p* upregulation, which inhibits M1 macrophage activation by targeting ERN1 (65).

Comprehensive miRNAomics analyses of cigarette smoke-exposed epithelial cell-derived exosomes identified 27 differentially expressed miRNAs, with *miR-21-3p* and *miR-27b-3p* promoting both M1 and M2 polarization through PI3K-AKT and MAPK pathways (66). Transcriptomic profiling revealed four genes (*NT5E, SDK1, TNS1, PCDH7*) with significant miRNA-mRNA interactions in COPD airway epithelium, particularly the *miR6511a-5p*-NT5E interaction relevant across multiple cell types including alveolar macrophages (68).

Emerging evidence links metabolic reprogramming to immune dysfunction. Bioinformatics analyses identified five ferroptosis-related hub genes (*HIF1A, IL6, PTGS2, CDKN1A, ATM*) that influence COPD pathogenesis, with immune profiling revealing upregulated monocytes and M0 macrophages alongside downregulated M2 macrophages (78). Similarly, anoikis resistance mechanisms involving TNMD and lncRNA 656 correlate with altered infiltration of activated CD4 memory T cells, M1 macrophages, and NK cells (77).

**Figure 2:**
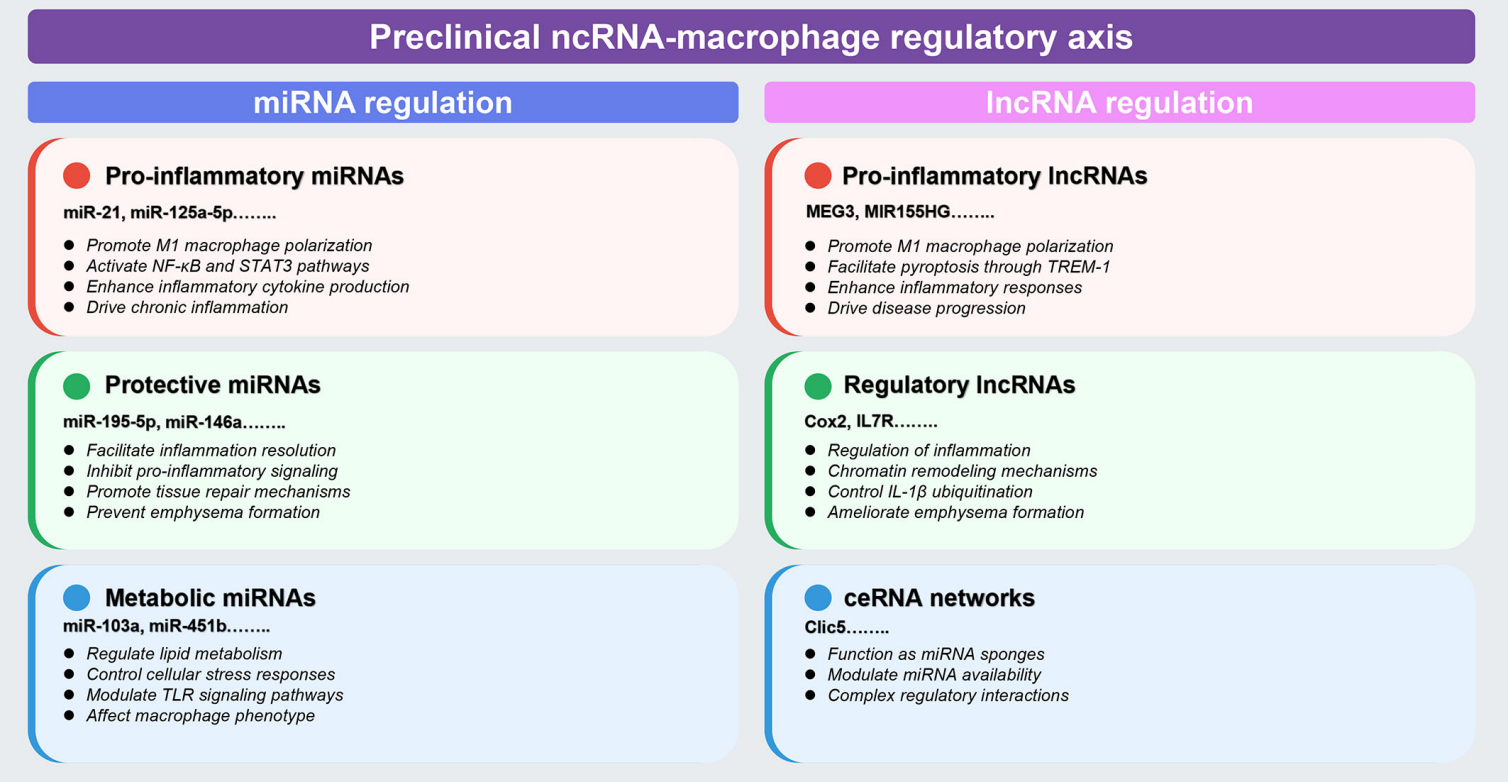
Preclinical landscape of ncRNA-macrophage regulatory axes in COPD. This comprehensive schematic synthesizes preclinical evidence from animal models and *in vitro* studies (detailed in **Tables 1**, **2**, Section 4.1). Color coding indicates functional categories: red boxes denote pro-inflammatory regulators, green boxes indicate protective/regulatory molecules, and blue boxes represent metabolic or ceRNA network components. Left panel (miRNA regulation): encompasses three major categories based on functional outcomes: (1) pro-inflammatory miRNAs (*miR-21, miR-125a-5p*, and others) that promote M1 macrophage polarization through NF-κB and STAT3 pathway activation; (2) protective miRNAs (*miR-195-5p, miR-146a*, and others) that facilitate inflammation resolution and prevent emphysema formation; and (3) metabolic miRNAs (*miR-103a, miR-451b*, and others) that regulate lipid metabolism and cellular stress responses. Right panel (lncRNA regulation): includes (1) pro-inflammatory lncRNAs (*MEG3, MIR155HG*, and others) that enhance inflammatory responses and drive disease progression through mechanisms including m6A methylation; (2) regulatory lncRNAs (*Cox2, IL7R*, and others) that control inflammation through chromatin remodeling and IL-1β ubiquitination; and (3) ceRNA networks (*Clic5* and others) that function as miRNA sponges to modulate regulatory interactions. Classification criteria are based on demonstrated effects on macrophage polarization, inflammatory cytokine production, and disease outcomes in experimental models. The figure was created using Figdraw (www.figdraw.com). ceRNA, competitive endogenous RNA; IL-1β, interleukin-1 beta; lncRNA, long non-coding RNA; miRNA, microRNA; ncRNA, non-coding RNA; NF-κB, nuclear factor-kappa B; STAT3, signal transducer and activator of transcription 3; TREM-1, triggering receptor expressed on myeloid cells-1.

Therapeutic translation has advanced through targeted delivery systems. Aspherical, nanostructured microparticles designed for siRNA delivery to macrophages achieved >30% TNF-α reduction in human macrophages, representing promising platforms for RNA therapy (79). These preclinical findings provide a robust mechanistic foundation for clinical investigation (**Figure 2**). The next section examines how these discoveries have been validated and extended in human COPD studies.

### 4.2 Clinical studies

Clinical investigations have successfully translated preclinical findings into human COPD contexts, establishing ncRNA-macrophage regulatory axes as clinically relevant mechanisms. These studies demonstrate three major translational achievements: (1) validation of global ncRNA dysregulation patterns in human alveolar macrophages; (2) identification of diagnostic biomarkers through ceRNA network analyses; and (3) discovery of novel RNA species that activate macrophage inflammatory responses. The following sections synthesize key clinical findings, with comprehensive details provided in **Table 3**.

**Table 3:** Clinical studies of ncRNAs and macrophage activation in COPD.

ncRNA/Regulatory network	Expression change	Clinical sample	Macrophage-related findings	Clinical significance	Diagnostic/Prognostic value	Reference
Global miRNAs	↓ in alveolar macrophages from smokers	Human alveolar macrophages	Inverse M1 mRNA expression program; decreased M1-induced transcripts, increased M1-repressed transcripts	Magnitude correlates with smoking history; many downregulated miRNAs target upregulated mRNAs	Potential for broad therapeutic targeting	(80)
LncRNA 482-*miR-6088*-PRRC2B ceRNA network	LncRNA 482 ↑, PRRC2B ↑ (positive correlation)	Peripheral blood mononuclear cells	Implications for COPD pathogenesis through post-transcriptional regulation	Demonstrates ceRNA network relevance in human disease	ROC analysis confirms diagnostic value	(81)
CircRNA/lncRNA-miRNA-mRNA networks	Complex dysregulation patterns	Lung tissues	↑M2 macrophages and activated NK cells in COPD tissues	TGF-β and Wnt/β-catenin pathways most dysregulated	18 hub genes identified for therapeutic targeting	(40)
SOCS3 and regulatory miRNAs	SOCS3+ ↑ in alveolar macrophages; *miR-19a-3p*, *miR-221-3p* ↑ in smokers without COPD	Human lung tissue and BAL	SOCS3+ alveolar macrophages higher in smokers; correlates with TNF-α+ macrophages and CD8+ T cell infiltration	SOCS3-suppressing miRNAs may represent protective mechanisms	CD14+SOCS3+ extracellular vesicles increased in COPD; potential inflammation biomarker	(82)
*miR-203a-3p*, *miR-375*	↑ in airways of smokers with and without COPD	Airway samples	*miR-203a-3p*: ↑proliferating-basal and secretory cells, ↓fibroblasts and immune cells; *miR-375*: altered endothelial, smooth muscle, mast cell proportions	Negative associations with pro-inflammatory pathways, positive with xenobiotic pathways	Potential biomarkers for smoking-related lung changes	(83)
*miR-29b*	*miR-29b* ↓ (due to DNA methylation), DNMT3A ↑	Human lung tissues	Negative feedback loop modulates M1 polarization and inflammation; affects Klotho methylation	Demonstrates methylation-mediated miRNA regulation in human COPD	Klotho promoter methylation as potential therapeutic target	(84)
*miR-93, miR-4454, miR-451a, miR-663a*	Divergent expression between upper and lower lung lobes	Alveolar macrophages and BAL fluid	Disparate miRNA expression in alveolar macrophages between lung regions; targets include cytokines and MMPs	Provides insights into upper lobe predominant disease patterns	Potential for understanding regional disease susceptibility	(85)
5’-tRNAValCAC half, rRNA-derived fragments	5’-tRNAValCAC half ↑ in COPD plasma; circulating rRNA fragments ↑	Plasma samples	5’-tRNAValCAC half activates macrophages via TLR7, induces cytokine production; rRNA fragments also induce macrophage cytokine production	Expands understanding of RNA-mediated inflammation beyond conventional ncRNAs	Novel inflammatory mediators with biomarker potential	(86)
Core miRNAs: *miR-543*, *miR-181c*, *miR-200a*	Identified through ML algorithms	Multiple datasets	Key immune cells: plasma cells, activated memory CD4 T cells, M2 macrophages identified	36 immune cell types show causal relationships with COPD	Diagnostic model development; 78 compounds and 437 traditional medicines predicted	(87)
*miR-34a-5p, miR-17-5p, miR-106a-5p, miR-20a-5p*	Expression shifts toward COPD patterns following CS exposure	Alveolar macrophages	Temporal progression analysis shows miRNA changes precede clinical disease	Provides insights into early molecular changes in disease development	Risk factors for COPD development identification	(88)

BAL, bronchoalveolar lavage; CD14, cluster of differentiation 14; ceRNA, competitive endogenous RNA; circRNA, circular RNA; CS, cigarette smoke; DNMT3A, DNA methyltransferase 3 alpha; lncRNA, long non-coding RNA; miR/miRNA, microRNA; ML, machine learning; MMPs, matrix metalloproteinases; mRNA, messenger RNA; ncRNAs, non-coding RNAs; NK, natural killer; PRRC2B, proline rich coiled-coil 2B; ROC, receiver operating characteristic; rRNA, ribosomal RNA; SOCS3, suppressor of cytokine signaling 3; TGF-β, transforming growth factor beta; TLR, toll-like receptor; TNF-α, tumor necrosis factor alpha; tRNA, transfer RNA; Wnt, wingless-related integration site; 5’-tRNAValCAC, 5’ half of tRNA valine with CAC anticodon; ↑, upregulated; ↓, downregulated.

#### 4.2.1 Global ncRNA expression patterns and ceRNA networks

Comprehensive clinical evidence confirms global miRNA downregulation in alveolar macrophages from smokers and COPD patients. Microarray analyses reveal an “inverse” M1 mRNA expression program, with decreased M1-induced transcripts and increased M1-repressed transcripts (80). Notably, the magnitude of global miRNA decrease correlates with smoking history, and downregulated miRNAs are predicted to target upregulated mRNAs, confirming miRNA-mediated post-transcriptional regulation in human disease (80).

Clinical validation of ceRNA networks has identified diagnostic biomarkers with translational potential. The lncRNA 482-*miR-6088*-PRRC2B ceRNA network demonstrates positive correlation between lncRNA 482 and PRRC2B expression in COPD patient peripheral blood mononuclear cells, with receiver operating characteristic analysis confirming diagnostic value (81). Systematic analyses of RNA transcripts identified 18 hub genes, revealing dysregulated TGF-β and Wnt/β-catenin signaling pathways, with increased M2 macrophages and activated NK cells in COPD lung tissues (40).

#### 4.2.2 Clinical biomarker development

Multiple biomarker discovery approaches have advanced clinical translation. SOCS3 regulation studies demonstrate significantly higher SOCS3^+^ alveolar macrophage percentages in smokers with and without COPD compared to non-smokers, correlating with TNF-α^+^ macrophages and CD8^+^ T cell infiltration (82). Importantly, CD14^+^SOCS3^+^ extracellular vesicles are increased in COPD patients, while SOCS3-suppressing miRNAs (*miR-19a-3p, miR-221-3p*) are elevated in smokers without COPD, suggesting protective mechanisms (82).

Large-scale expression analyses identified smoking-associated miRNA signatures. High *miR-203a-3p* and *miR-375* expression in smokers’ airways demonstrate negative associations with pro-inflammatory pathway genes and positive associations with xenobiotic pathway genes (83). Cellular deconvolution analyses reveal that these miRNAs correlate with altered cellular proportions, including immune cells, fibroblasts, and smooth muscle cells (83).

Epigenetic mechanisms provide additional regulatory insights. *miR-29b* downregulation due to increased DNA methylation in COPD patient lung tissues reveals a negative feedback loop with DNMT3A that modulates cigarette smoke-induced M1 polarization (84). Regional expression analyses demonstrate divergent miRNA patterns between upper and lower lung lobes, with altered *miR-93*, *miR-4454*, *miR-451a*, and *miR-663a* expression in alveolar macrophages and bronchoalveolar lavage fluid, potentially explaining upper lobe predominance (85).

#### 4.2.3 Novel RNA species and predictive models

Clinical discoveries have expanded beyond conventional ncRNAs. Immunoactive signatures of circulating tRNA- and rRNA-derived RNAs reveal remarkable accumulation of 5’-tRNAValCAC half in COPD patient plasma, which activates human macrophages via TLR7 and induces cytokine production (86). Circulating rRNA-derived fragments similarly induce macrophage cytokine production, suggesting novel inflammatory mediators (86).

Machine learning approaches have enhanced translational potential. Algorithms predicted core miRNAs (*hsa-miR-543, hsa-miR-181c, hsa-miR-200a*), key immune cells (plasma cells, activated memory CD4 T cells, M2 macrophages), and characteristic genes (*EGF, PLG, PTPN22, NR4A1*) associated with COPD (87). Mendelian randomization analysis revealed causal relationships between 36 immune cell types and COPD, highlighting central immune dysfunction (87). Temporal progression studies demonstrate that specific miRNA signatures (*hsa-miR-34a-5p, miR-17-5p, miR-106a-5p, miR-20a-5p*) shift toward COPD patterns following cigarette smoke exposure, identifying early risk factors (88).

**Figure 3:**
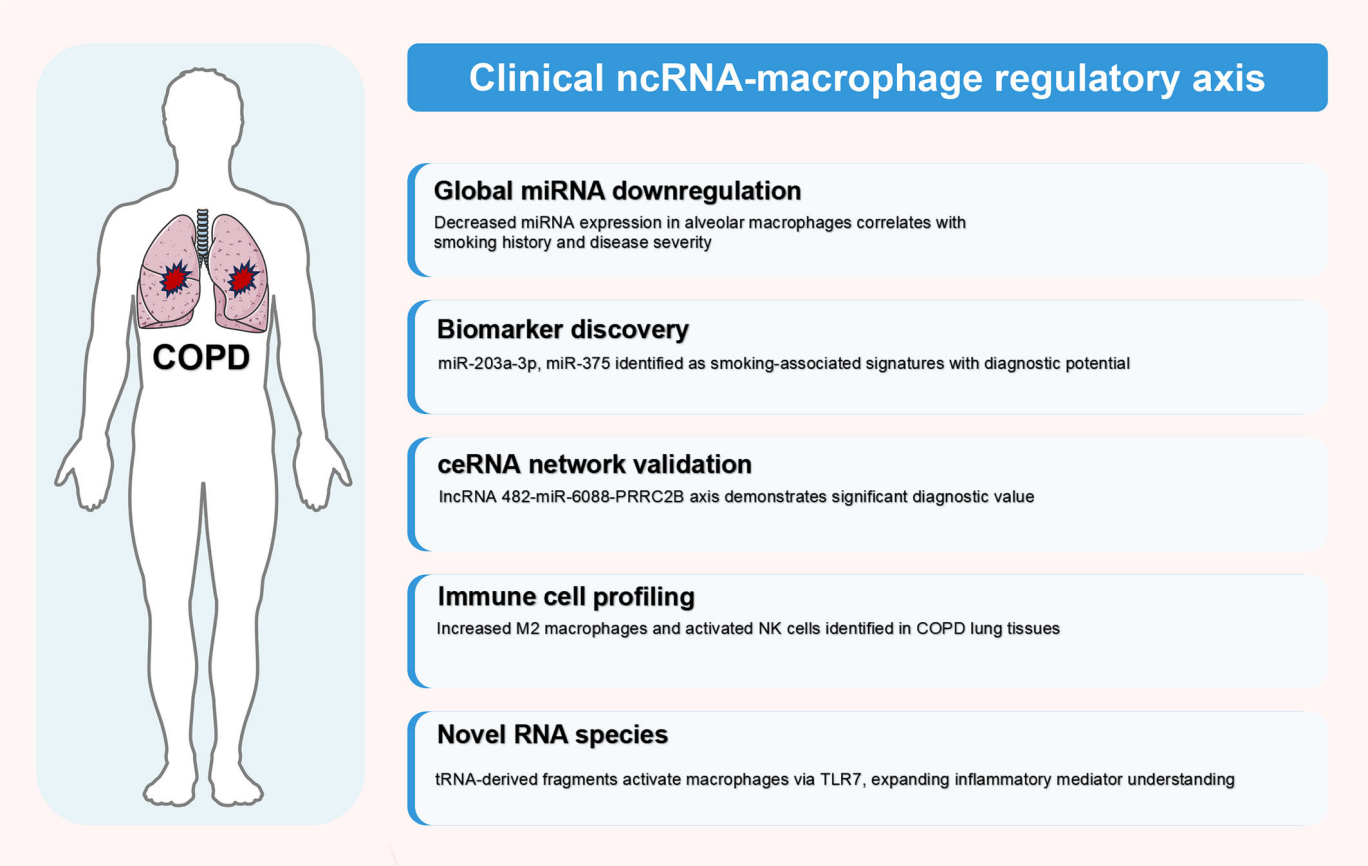
Clinical validation of ncRNA-macrophage regulatory axes in human COPD. This figure illustrates clinical translation of ncRNA-macrophage regulatory mechanisms in human COPD patients, synthesizing findings from clinical studies detailed in **Table 3** (Section 4.2). Evidence is categorized into five hierarchical domains based on translational relevance: global miRNA downregulation, biomarker discovery, ceRNA network validation, immune cell profiling, and novel RNA species identification. These clinical discoveries provide crucial validation of preclinical findings and establish the therapeutic potential of targeting ncRNA-macrophage regulatory axes in COPD management. The figure was created using Figdraw (www.figdraw.com). ceRNA, competitive endogenous RNA; COPD, chronic obstructive pulmonary disease; miRNA, microRNA; ncRNA, non-coding RNA.

These clinical investigations successfully validate preclinical mechanisms while establishing ncRNA-macrophage regulatory axes as clinically actionable targets. Human studies confirm global miRNA dysregulation, validate ceRNA networks with diagnostic potential, identify novel RNA species activating macrophages, and develop predictive models for disease progression. The integration of omics technologies, machine learning, and longitudinal analyses provides crucial validation for therapeutic development. Additional clinical findings and their translational implications are detailed in **Table 3**. The comprehensive clinical evidence, illustrated in **Figure 3**, supports advancing ncRNA-based precision medicine approaches for COPD management.

In summary, the extensive body of preclinical and clinical research has established ncRNA-macrophage regulatory axes as fundamental components of COPD pathogenesis. Preclinical studies have identified specific miRNAs, lncRNAs, and their downstream targets that control macrophage polarization, inflammatory responses, and tissue repair mechanisms, with clear dichotomous patterns of pro-inflammatory versus protective regulatory networks. Clinical investigations have successfully validated these mechanistic findings in human disease, demonstrating altered ncRNA expression patterns in patient samples that correlate with disease severity, exacerbation frequency, and therapeutic responses. The identification of novel RNA species, including tRNA-derived fragments, and the application of machine learning approaches have expanded our understanding beyond traditional ncRNA categories. The convergence of preclinical mechanistic insights with clinical validation provides a robust foundation for developing ncRNA-based diagnostics and therapeutics, though significant challenges remain in achieving cell-type specificity and overcoming delivery barriers. These findings collectively underscore the translational potential of targeting ncRNA-macrophage regulatory axes for precision medicine approaches in COPD management.

## 5 Systematic evaluation of current research: challenges and limitations

While both preclinical and clinical studies have significantly advanced our understanding of ncRNA-macrophage regulatory axes in COPD, critical evaluation of the current evidence reveals important methodological limitations and knowledge gaps that must be addressed.

### 5.1 Research progress and methodological advances

Current research on ncRNA-macrophage regulatory axes in COPD has demonstrated substantial methodological sophistication, particularly through the integration of multi-omics approaches with functional validation studies. Single-cell RNA sequencing technologies have enhanced understanding of macrophage heterogeneity within the COPD lung microenvironment (80), while sophisticated bioinformatics pipelines for ceRNA network construction have enabled accurate prediction and validation of complex regulatory interactions (40, 81).

Clinical translation efforts have yielded encouraging results in biomarker development and therapeutic target identification. The validation of specific miRNA signatures in large patient cohorts has demonstrated robust diagnostic and prognostic potential (83, 87), while the successful development of targeted delivery systems represents significant progress toward clinical implementation (79).

### 5.2 Critical limitations and knowledge gaps

#### 5.2.1 Experimental design constraints and methodological heterogeneity

Despite significant progress, several fundamental limitations persist. The predominant reliance on cigarette smoke exposure models may not fully capture COPD complexity, particularly in patients with biomass exposure or genetic susceptibility factors. Additionally, the heterogeneity in macrophage isolation and characterization protocols across studies has introduced substantial variability in findings (40, 80–82).

Current isolation methods vary significantly: bronchoalveolar lavage yields primarily alveolar macrophages but may induce mechanical activation, while enzymatic digestion protocols use diverse conditions (collagenase: 0.5–2 mg/mL, elastase: 200–400 U/mL, Liberase™: 100-400 μg/mL, 30–90 minutes). Liberase™ provides superior interstitial macrophage yield, particularly for IM3 subsets, though elastase causes selective marker loss including CD64 and MerTK (89–91). Flow cytometry panels range from simple two-color staining to 12-parameter analyses, affecting M1/M2 classification reliability (53, 70).

The lack of standardized criteria for defining M1 and M2 polarization states, particularly in chronic inflammatory diseases, has complicated result interpretation and cross-study comparisons. Furthermore, conventional *in vitro* polarization protocols may inadequately reflect the complex, mixed activation states observed in human COPD lung tissue (70, 72).

#### 5.2.2 Technical and clinical translation barriers

Current analytical approaches face technical limitations including variable sensitivity and specificity of ncRNA detection methods between platforms, leading to inconsistent results. Computational prediction algorithms for ncRNA target identification suffer from high false-positive rates requiring extensive experimental validation (66–68). Species-specific differences in ncRNA sequences and expression patterns between rodent models and humans pose substantial challenges for direct translation (50, 51, 67). Moreover, the complex pharmacokinetics and biodistribution of ncRNA-based therapeutics remain poorly understood, particularly regarding stability and delivery to specific lung cell populations.

The therapeutic delivery of ncRNA-based interventions represents a paramount barrier to clinical translation in COPD. Current delivery platforms each present distinct advantages and critical limitations. Lipid nanoparticles (LNPs), while clinically validated for systemic delivery, face challenges in pulmonary applications including rapid alveolar macrophage clearance, limited mucus penetration, and lack of cell-type specificity (92). Polymeric nanoparticles offer improved stability but suffer from size-dependent mucus barrier limitations and potential immunogenicity (93). Exosome-based systems provide natural biocompatibility but are hampered by low cargo loading efficiency, manufacturing standardization challenges, and heterogeneous populations (94). The study by Fischer et al. (79) achieved only 30% TNF-α knockdown using microparticulate siRNA delivery, highlighting the gap between current capabilities and the >70% suppression typically required for therapeutic efficacy. Inhaled formulations, while enabling local delivery with reduced systemic exposure, face stability challenges during aerosolization and impaired distribution due to COPD-associated mucociliary dysfunction (95).

Specific barriers further complicate clinical translation. RNA stability requires chemical modifications (2’-O-methyl, phosphorothioate) that may compromise specificity and increase toxicity (96). Targeting specificity remains suboptimal despite ligand-based approaches (mannose, antibodies), with the heterogeneity of lung macrophage populations requiring subset-specific strategies (97). Off-target effects persist even with local delivery, as partial complementarity and TLRs activation can trigger unintended responses (98). Among current technologies, LNPs are closest to clinical implementation with several candidates in respiratory disease trials, yet no inhaled ncRNA therapeutic has achieved regulatory approval for COPD (99). Future advances require integration of smart nanoparticles with stimuli-responsive release, biomimetic coatings for enhanced biocompatibility, and combination delivery strategies targeting multiple regulatory nodes simultaneously (100).

#### 5.2.3 Clinical heterogeneity and mechanistic understanding

Current research has largely treated COPD as a homogeneous condition, despite growing recognition of distinct disease endotypes with different molecular characteristics. The extent to which ncRNA-macrophage regulatory patterns vary between different COPD phenotypes remains unclear (77, 78, 101).

Furthermore, the understanding of how individual ncRNA-macrophage pathways integrate into comprehensive regulatory networks remains limited. The temporal dynamics of network activation and the hierarchical organization of regulatory controls require further investigation (69, 75). Additionally, a fundamental limitation that has received insufficient attention is the heterogeneous nature of lung macrophage populations and their distinct roles in COPD pathogenesis.

#### 5.2.4 Macrophage heterogeneity: a critical gap in current ncRNA research

Current ncRNA-macrophage research in COPD inadequately addresses the fundamental heterogeneity of pulmonary macrophage populations, representing a critical limitation affecting research interpretation and therapeutic development. Lung macrophages comprise anatomically and functionally distinct subsets: AMs residing in airspaces and IMs within lung parenchyma (102). AMs serve as sentinel cells for pathogen recognition and particle clearance, while IMs primarily mediate tissue remodeling and stromal interactions (5). In COPD, these populations exhibit distinct dysfunction patterns and molecular signatures that may respond differently to ncRNA regulatory networks (5, 90).

Additionally, developmental origin creates further complexity, with tissue-resident macrophages (embryonic-derived, self-renewing) displaying different transcriptional programs compared to recruited monocyte-derived macrophages that infiltrate during inflammation (103). COPD significantly alters this balance, increasing monocyte recruitment and potentially creating distinct ncRNA expression profiles between these populations (5, 6).

The failure to distinguish between macrophage subsets in current studies may mask cell type-specific regulatory networks, explain apparent contradictions in research findings, and limit therapeutic efficacy (104). Future research should employ single-cell RNA sequencing and subset-specific approaches to characterize ncRNA regulatory networks within defined macrophage populations, enabling development of precision therapeutic strategies targeting specific pathogenic mechanisms while preserving beneficial macrophage functions.

#### 5.2.5 Experimental validation strategies for ceRNA networks

The computational prediction of ceRNA networks, while powerful, requires rigorous experimental validation to establish functional relevance in COPD pathogenesis. Multiple complementary approaches have been developed to validate these regulatory interactions, each providing distinct insights into network functionality.

Knockdown and overexpression experiments represent the foundational approach for ceRNA network validation. In COPD research, loss-of-function studies using siRNAs or short hairpin RNAs targeting specific lncRNAs or circRNAs can demonstrate their regulatory effects on miRNA availability and downstream target gene expression (66, 67, 70, 71). For instance, lncRNA-*Clic5* plays a ceRNA regulatory role by sponging *miR-212-5p* to attenuate the regulation of RASSF1. Moreover, lncRNA*-Clic5* overexpression inhibited rat alveolar macrophages apoptosis by targeting the *miR-212-5p*/RASSF1 pathway. Co-treatment with *miR-212-5p* and lncRNA*-Clic5* in the presence of cow barn PM2.5 revealed that lncRNA*-Clic5* reversed rat alveolar macrophages cell apoptosis induced by PM2.5 when *miR-212-5p* was overexpressed (76).

Dual-luciferase reporter assays provide direct evidence for miRNA-target binding within ceRNA networks. This approach involves cloning predicted miRNA binding sites from lncRNAs, circRNAs, or target mRNAs into luciferase reporter vectors (105). Co-transfection with miRNA mimics should result in decreased luciferase activity, while mutation of binding sites should abolish this effect. In COPD ceRNA research, this method has been crucial for validating interactions such as the lncRNA *MIR155HG*-miRNA-target networks that regulate macrophage polarization (70, 71).

RNA immunoprecipitation assays enable detection of RNA-protein and RNA-RNA interactions within endogenous cellular contexts. Using antibodies against Argonaute proteins, researchers can precipitate miRNA-containing RNA-induced silencing complexes and identify co-precipitated lncRNAs, circRNAs, and mRNAs (106). This technique has proven particularly valuable for validating ceRNA networks in primary alveolar macrophages from COPD patients, where artificial overexpression systems may not accurately reflect physiological conditions (107).

Fluorescence *in situ* hybridization and proximity ligation assays provide spatial validation of ceRNA interactions within specific cell types and subcellular compartments. In COPD research, these techniques have been particularly useful for confirming co-localization of interacting RNAs in alveolar macrophages and epithelial cells (60, 108).

These diverse methodologies provide robust frameworks for the systematic investigation and validation of ceRNA networks in COPD, thereby strengthening the methodological rigor and reliability of related research. As technological capabilities continue to advance, more comprehensive analyses of ceRNA networks are expected, enabling deeper mechanistic understanding and uncovering novel molecular insights into the pathogenesis and progression of COPD.

### 5.3 Quality assessment and reproducibility concerns

Systematic assessment reveals significant variations in study quality and reporting standards. While some investigations demonstrate rigorous experimental design, others suffer from inadequate sample sizes, lack of proper validation, and insufficient characterization of experimental conditions (86, 88).

The field may be subject to publication bias, with negative results underrepresented in the literature, potentially overestimating therapeutic potential while underestimating clinical translation challenges. Additionally, reproducibility concerns persist due to variations in experimental protocols, reagent sources, and analytical methods across different laboratories (66–68).

Current ncRNA-macrophage research in COPD suggests substantial mechanistic progress yet faces significant challenges in standardization, clinical translation, and comprehensive network understanding. Addressing these limitations through standardized protocols, physiologically relevant models, and systematic approaches to identified knowledge gaps will be crucial for realizing therapeutic potential.

While substantial progress has been made in elucidating ncRNA-macrophage regulatory networks in COPD, critical limitations persist that must be addressed to realize therapeutic potential. The field faces significant methodological challenges including heterogeneous experimental protocols, inadequate representation of macrophage subset diversity, and limited understanding of integrated regulatory networks. The predominant use of cigarette smoke models may not capture the full spectrum of COPD phenotypes, particularly those related to biomass exposure or genetic susceptibility. Technical barriers such as species-specific ncRNA differences, computational prediction accuracy, and delivery system optimization remain substantial obstacles to clinical translation. Perhaps most critically, the failure to adequately address macrophage heterogeneity—including distinct anatomical populations and developmental origins—represents a fundamental gap that may explain conflicting findings and limit therapeutic efficacy. Addressing these challenges through standardized protocols, advanced single-cell technologies, and comprehensive network analyses will be essential for advancing the field toward clinical implementation.

## 6 Future research directions and perspectives

Addressing the identified limitations requires strategic deployment of emerging technologies and innovative experimental approaches. The following priorities will accelerate translation of ncRNA-macrophage research into clinical applications.

### 6.1 Technological innovation

The integration of single-cell multi-omics technologies with spatial transcriptomics will provide unprecedented resolution of ncRNA-macrophage interactions within the COPD lung microenvironment. Machine learning approaches will accelerate ncRNA-target interaction prediction and enable development of predictive models for therapeutic responses (40, 68). Human lung organoids derived from patient-specific induced pluripotent stem cells will provide physiologically relevant platforms that accounting for genetic diversity and disease heterogeneity (109).

### 6.2 Therapeutic development

Future strategies will embrace precision medicine through companion diagnostics based on ncRNA expression profiles, enabling patient stratification for optimal interventions (83, 87). Combination therapy approaches targeting multiple ncRNA pathways simultaneously will enable comprehensive macrophage function modulation, while advanced delivery systems will achieve cell-type-specific targeting with improved stability (35, 79). Particularly, miRNAs represent attractive therapeutic targets due to their small size, sequence-specific targeting, and critical roles in disease pathogenesis. Emerging platforms include miRNA-derived oligonucleotide therapeutics and lipid nanoparticle-based delivery systems for miRNA-derived chemically modified nucleoside drugs, demonstrating enhanced stability and cellular uptake (110, 111).

### 6.3 Clinical implementation

Multicenter validation studies with standardized protocols will establish diagnostic and prognostic value of ncRNA signatures (81, 86). Development of point-of-care diagnostic devices will facilitate precision medicine implementation in routine practice. Regulatory framework development and economic evaluations will be essential for successful clinical translation of ncRNA-based therapeutics.

The future of ncRNA-macrophage research in COPD lies at the intersection of technological innovation, precision medicine, and systems biology approaches. Integration of single-cell multi-omics with spatial transcriptomics and machine learning will enable unprecedented resolution of cellular heterogeneity and regulatory networks within the lung microenvironment. The development of advanced delivery systems, including cell-type-specific nanoparticles and engineered exosomes, coupled with combination therapeutic strategies targeting multiple regulatory nodes, holds promise for overcoming current translational barriers. The implementation of companion diagnostics based on ncRNA signatures will facilitate patient stratification and personalized treatment selection. Success will require coordinated efforts across disciplines, including standardization of research protocols, establishment of robust biomarker validation frameworks, and development of appropriate regulatory pathways for ncRNA-based therapeutics. These advances position the field for transformative breakthroughs that could fundamentally alter COPD management, moving from symptomatic treatment toward disease-modifying interventions that target the underlying molecular pathophysiology.

## 7 Conclusion

This comprehensive review has elucidated the pivotal role of ncRNA-macrophage regulatory axes in COPD pathogenesis. Our analysis reveals a dichotomous regulatory pattern. Pro-inflammatory miRNAs (*miR-21, miR-125a-5p, miR-221-3p*) promote pathological M1 macrophage polarization through NF-κB and STAT3 signaling pathways, while protective miRNAs (*miR-195-5p, let-7c, miR-146a*) facilitate inflammation resolution and tissue repair (14, 15, 49, 52–54). LncRNAs demonstrate sophisticated regulatory mechanisms through transcriptional scaffolding and ceRNA networks, with molecules such as *MEG3* and *MIR155HG* driving inflammatory responses (69, 70, 74, 75).

Clinical validation studies have successfully translated these mechanistic insights into human disease contexts, establishing ncRNA signatures as potential diagnostic biomarkers and identifying actionable therapeutic targets (40, 80–84). The discovery of dysregulated ncRNA expression patterns in COPD patients, coupled with their correlation with disease severity and progression, supports the development of precision medicine approaches. Notably, the therapeutic potential of targeting ncRNA-macrophage regulatory axes has been demonstrated through successful modulation of macrophage polarization and inflammatory responses in preclinical models, offering promising avenues for combination therapeutics.

Despite significant progress, several challenges must be addressed to realize the full therapeutic potential of ncRNA-based interventions. These include methodological standardization across studies, species-specific differences limiting clinical translation, and the need for advanced delivery systems to achieve cell-type-specific targeting (67, 79, 80). Future research should prioritize the integration of single-cell multi-omics technologies with spatial transcriptomics to better understand ncRNA-macrophage interactions within the lung microenvironment. The development of companion diagnostics based on ncRNA expression profiles will enable patient stratification for personalized therapeutic interventions. Ultimately, continued interdisciplinary collaboration between researchers, clinicians, and regulatory experts will be essential for translating these promising findings into clinical practice and improving outcomes for COPD patients worldwide.
